# A national strategy to engage medical students in otolaryngology-head and neck surgery medical education: the LearnENT ambassador program

**DOI:** 10.1007/s40037-020-00607-y

**Published:** 2020-08-14

**Authors:** Marc Levin, Elysia Grose, Corliss Best, Scott Kohlert

**Affiliations:** 1 Department of Otolaryngology—Head and Neck Surgery, University of Toronto, Toronto, Ontario Canada; 2 University of Ottawa Faculty of Medicine, University of Ottawa, Ottawa, Ontario Canada; 3 Department of Otolaryngology—Head and Neck Surgery, University of Ottawa, Ottawa, Ontario Canada; 4 ENT Associates of East Texas, Tyler, Texas USA

**Keywords:** Undergraduate medical education, Otolaryngology-Head and Neck Surgery, e‑Learning, Mobile-app

## Abstract

**Background:**

In the realm of medical education, student-led ambassador programs represent an innovative approach to increase awareness about medical education resources. LearnENT is an internationally recognized otolaryngology-head and neck surgery (OHNS) smartphone app and website designed for medical trainees to learn about OHNS. However, upon the initial launch of the app, there was a lack of medical student awareness and engagement.

**Approach:**

In this article, we highlight the process and lessons learned from developing an ambassador program to increase the national presence and uptake of LearnENT. Medical students from across Canada were recruited and trained to promote the app at their respective institutions.

**Evaluation:**

Ambassadors hosted events and spearheaded initiatives around the country with the goal of showcasing LearnENT. Furthermore, ambassadors were engaged in scholarly initiatives such as creating educational content for LearnENT and giving presentations at national conferences.

**Reflections:**

Critical factors in the success of a student-led ambassador program include ensuring widespread dissemination of the program, establishing clear expectations for ambassadors, equipping ambassadors with standardized promotional material, and promoting collaboration to collectively work towards addressing challenges. When creating a national student-led group such as an ambassador program, outreach to senior stakeholders can be an effective way to involve students at different institutions, provide mentorship opportunities for students and provide opportunities for educational scholarship. With new medical education innovations constantly surfacing, the LearnENT ambassador program model can be applied in other contexts to increase awareness of medical education resources.

## Background and need for innovation

Undergraduate medical education in otolaryngology-head and neck surgery (OHNS) is highly variable in Canada [1, 2]. One study from a Canadian medical school demonstrated that approximately 70% of medical students had never observed an otolaryngologist-head and neck surgeon. Furthermore, over 90% reported very little OHNS exposure during medical school [3]. Thus, there is a need for standardizing and improving undergraduate medical education in OHNS. LearnENT is an educational smartphone app and website initially designed at the University of Ottawa, which is aimed at bridging the gap in OHNS medical education [4]. This app provides medical students, residents, and physicians with up-to-date, evidence-based, interactive learning material covering a breadth of OHNS topics. To date, LearnENT is the official learning app of the Canadian Society of Otolaryngology-Head and Neck Surgery (CSOHNS) and remains the first known free, public, app-based education platform fully endorsed by a national surgical society. When the app launched its most recent version in 2018, several of the faculty members involved in the development of LearnENT promoted the app at their respective institutions. However, while LearnENT was created for learners, there was a lack of medical student awareness and involvement in the app.

The social learning theory suggests that new behaviors can be acquired through observing and imitating the behavior of others [5]. When applied to medical education, the social learning theory can be used as a basis to increase the usage and awareness of medical education resources. Hence, if medical students use and promote a resource to their colleagues, they may be more likely to adopt the resource and learn its material. Brand ambassador programs have been widely used in the marketing industry and apply several of the principles underlying the social learning theory [5]. Ambassador programs utilize influential representatives to increase product awareness and advocate for widespread use of the product [6, 7]. Thus, it stands to reason that medical student ambassador programs can be effectively utilized to promote medical education resources to their colleagues. Medical student-lead endeavors, such as interest groups and educational initiatives, have been shown to be successful in fostering interest among fellow students [8, 9]. Furthermore, there is plenty of evidence to suggest that increasing learner engagement in medical education initiatives promotes the development of core physician competencies outlined by CanMEDS [10, 11]. Thus, a brand ambassador program was created to increase medical student engagement with LearnENT, which was grounded in the principles of the social learning theory.

In this paper, the steps taken to develop and implement a national ambassador program for a medical education smartphone app are outlined. This paper also reflects upon the challenges and successes encountered throughout this process. The development of this program can serve as a framework to be replicated or adapted for other educational initiatives.

## Goals of innovation

With an aim to increase the outreach and uptake of LearnENT on a national level by medical learners, a student-led LearnENT Ambassador Program (LAP) was inaugurated in August of 2018. The main goals of the LAP were twofold: 1) to promote the regular use of the LearnENT app among medical trainees across Canada and 2) to encourage leaders in OHNS medical education to contribute content and ideas to the app. With this aim, it was decided that the most appropriate ambassadors would be medical students. As the app was designed primarily for undergraduate medical education, medical student representatives were more likely to use the app and would be more influential in promoting the app to their peers. Medical students also have ample ability to host events and disseminate information to the app’s target audience through various already created student groups. Furthermore, it was felt that medical students would benefit the most from this role as it had the potential to bring about several mentorship and networking opportunities. The main roles of a LearnENT Ambassador include:

- Hosting interactive presentations and events about LearnENT at their respective institutions
- Engaging staff, residents and students to contribute to LearnENT
- Acting as liaison between staff, residents and medical students to provide feedback to the LearnENT team
- Developing innovative ways to increase the awareness of LearnENT and incorporate LearnENT into their OHNS curriculum

## Steps taken for development and implementation of innovation

### Framework for developing an ambassador program

**Fig. 1:**
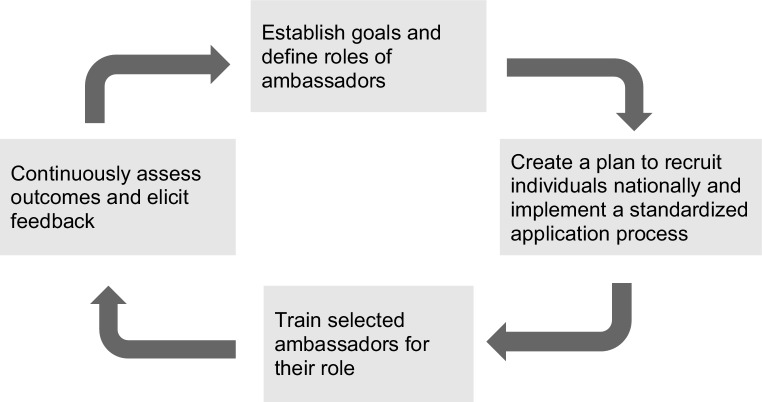
An overview of the steps taken to successfully develop and implement an ambassador program

In the technology innovation sector, ambassador programs have been widely used by successful companies such as Xbox, Adobe and more [12]. Previously developed ambassador programs were investigated to form the foundations for the LAP. Some key characteristics identified for the implementation of a successful ambassador program include formulating clear and measurable goals for the program [6]. Other successful ambassador programs have incorporated structured processes to select and train ambassadors [6]. Furthermore, previous ambassador programs have established that both vertical and horizontal communication is necessary to maintain motivation, continuously assess the outcomes of the program, and elicit feedback about the program (Fig. 1; [6]).

### Implementation of the LAP

Once the goals of the LAP were established, nationwide advertisements about LAP applications for medical students were initiated by the two medical student co-directors of the LAP (M.L. and E. G.). The advertisements included the roles and responsibilities of a LearnENT ambassador. As a way to provide incentive to medical students, advertisements included information about the potential networking, mentorship, and scholarship opportunities available through this role. Advertisements were focused on recruiting first and second year medical students; however, no restrictions based on year of study were placed. The reasoning behind primarily recruiting students in their initial years of medical school was twofold. At the majority of medical schools in Canada, student-led events and interest groups are more likely to be attended by students in their pre-clerkship years. Additionally, this recruitment strategy was implemented to ensure longevity of the program as students in their early years were likely to continue with this role for consecutive years until graduation. Medical school student societies, residents and staff physicians across Canada were involved in advertising applications for the LAP. Additionally, LAP applications were advertised on social media and at the Annual General Meeting for the CSOHNS. Medical students subsequently submitted written applications to the LAP which included basic questions regarding the applicant’s interest in OHNS, motivation to become a LearnENT ambassador, and any previous leadership experience. Applicants were selected for the LearnENT ambassador role based on the quality of their answers to the written application and on their previously demonstrated ability to take initiative, implement successful events and engage in innovation.

Following written applications, ambassadors participated in a teleconference with the two co-directors. The goals of these teleconference meetings included getting to know the applicants, discussing the roles and responsibilities of ambassadors and organizing action plans for each ambassador at their respective schools. Discussing action plans included providing examples of potential events, inquiring about existing student-led groups which could help endorse LearnENT, discussing the ambassador’s school’s OHNS curriculum and opportunities for LearnENT curriculum involvement. This process helped us identify unique barriers specific to each school and help the ambassador work around them. For instance, some schools have multiple campuses and thus, enabling students to join in virtually was suggested in these instances. Additionally, some schools did not have an OHNS residency program and thus, it was not possible to involve residents in the events. In such cases, the LAP co-directors helped students connect with staff members at their institutions who would be open to participating in LAP events and brainstorm other purely student-led event ideas.

**Fig. 2:**
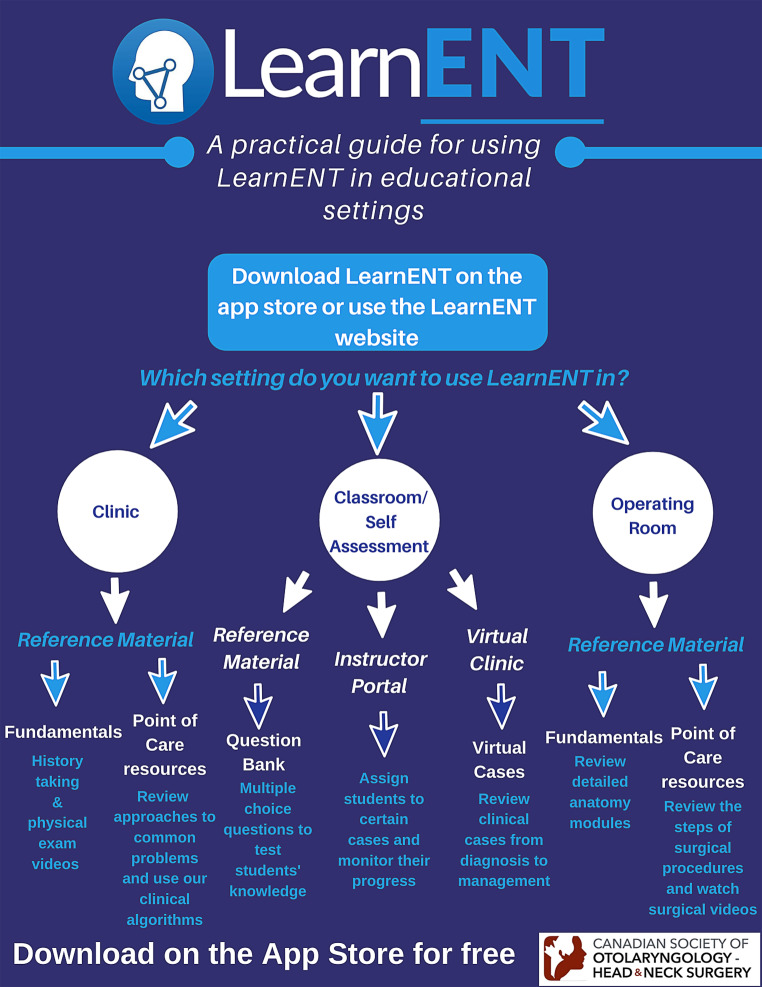
LearnENT Poster

After this teleconference, each ambassador was trained in the various features of the app and coached on appropriate ways to promote the app at their events. Each ambassador was also equipped with standardized resources including LearnENT information posters, promotional videos, email templates, and PowerPoint slides (Fig. 2). These materials were intended to be distributed by the ambassadors at their institutions. Throughout the year, ambassadors were continuously updated about new opportunities and ways to contribute to the LAP through email. Nationwide teleconference meetings were held where all ambassadors shared innovative ideas, discussed any challenges they encountered and brainstormed future ideas for the LAP. As arranging a time when each member could meet was often a challenge, meeting minutes were taken and distributed to ambassadors following the meetings. The progress of the LAP was tracked by requiring ambassadors to provide email updates about their initiatives at their respective schools and through discussion at the national meetings.

## Outcomes of innovation

Since the initiation of the LAP in August 2018, ten out of the seventeen Canadian medical schools currently have LearnENT ambassadors. Ambassadors at all ten schools across Canada have planned events aimed at increasing the uptake of LearnENT. For example, at the University of Ottawa, University of Toronto, University of Saskatchewan and Western University, the ambassadors hosted events showcasing LearnENT and highlighting specific features of the app such as the virtual clinic, physical exam videos, and question banks. These events were often combined with discussions hosted by OHNS residents and staff physicians about OHNS as a career choice. At McMaster University, the LearnENT ambassadors hosted an interactive event where an OHNS resident reviewed a case they had written for LearnENT with a group of students. Furthermore, LearnENT was presented at McGill University at the medical school’s annual academic fair where products specifically designed for medical students are showcased. In addition, ambassadors across the country have facilitated endorsement of the app through student societies circulating the LearnENT promotional materials to medical students.

The LAP has also helped to advertise LearnENT in the classroom and clinical setting with several educators in OHNS including advertisements for LearnENT in their lectures. Ambassadors have been integral in initiating discussions with their faculties about integrating LearnENT into the OHNS curricula and several schools have incorporated the learning material in LearnENT into their virtual curricula. For example, the University of Ottawa has incorporated LearnENT cases as a  component of the OHNS clerkship curriculum.

The ambassadors have also helped contribute to the educational material on the app. Since the implementation of the LearnENT ambassador program, nine new learning cases have been written and uploaded to the app, several of which were written by ambassadors in collaboration with faculty members at their respective institutions. Furthermore, the LAP has allowed ambassadors to present and host workshops about LearnENT at national meetings such as the annual meeting for the CSOHNS.

## Critical reflection

Overall, the LAP has been instrumental in extending the national presence of LearnENT and for expanding medical education in OHNS. Moreover, this program has created valuable leadership and networking opportunities for the involved students. Both the successes and challenges associated with the development of the LAP are worth reflecting on to provide insight to other leaders in medical education hoping to adopt a similar process. Below, the authors highlight key lessons learned about the development and implementation of a student-led ambassador program.

1. *Continuous student outreach promotes widespread engagement and accessibility*

In order to promote the LAP, the co-founders reached out to schools across Canada through various avenues including their student societies, social media, and advertising the program at national OHNS meetings. Through these avenues, the program reached the majority of medical schools across Canada. Despite this, some institutions do not have LearnENT ambassadors. Since the LAP co-founders relied heavily on student representatives from each institution to disseminate LAP applications, some institutions lacked easily reachable contacts. Since both the LAP co-founders were based at an Ontario medical school, there were many pre-existing connections to other Ontario schools which enabled ample advertisement of the program. Thus, the ambassadors at different institutions across the country, particularly those from Eastern and Western Canada, have been resourceful in connecting with their neighboring schools to increase the uptake of this program. Another ongoing challenge is the lack of ambassador representation in the francophone medical schools. Thus, the co-founders of the program continue to ensure the LAP is accessible to all medical students in Canada by having French-speaking medical students advertising the program to francophone medical schools.

2. *Equip ambassadors with standardized tools to meet program expectations*

Ideas for ambassador-led initiatives were brainstormed individually with each ambassador in collaboration with the two co-directors during the initial teleconference meeting. This helped set expectations for ambassador-led initiatives and encouraged students to think about how they can best integrate LAP initiatives at their specific institutions. In doing so, school-specific events were created that were relevant and tailored best to their own students, residents and faculty. With this autonomy, however, ambassadors were equipped with standardized LearnENT posters, lecture slides, videos, and email templates that they could then creatively share with their own schools. The lecture slides were particularly effective as students could simply send these to their professors to include them in their lecture slides. Furthermore, the posters were distributed through email and at national meetings and served as effective visual guides on how to best use the app in different settings. This helped ensure both an adequate amount of standardization among ambassador-led initiatives across the country in addition to allowing for flexibility and creativity.

3. *Liaison with faculty and senior stakeholders builds uptake and promotes scholarship and mentorship*

Faculty members and resident physicians were integral in promoting the uptake of the LAP. The wide majority of institutions that currently have LearnENT ambassadors have faculty members dedicated to the promotion of medical education initiatives who advocated to get students at their respective institutions involved in the LAP. Faculty have also been instrumental in helping LearnENT ambassadors integrate LearnENT into medical school curricula. In addition to promoting the uptake of LearnENT, the LAP served to provide students with additional opportunities to explore their interest in and contribute to OHNS medical education. Through notifying residents and faculty members about the LAP, opportunities were created for students to collaborate with them to create content for LearnENT. Furthermore, several residents and faculty members assisted in leading the LAP events at various institutions which helped provide networking opportunities not only for the ambassadors but also other students attending the events.

4. *Collaboration ensures challenges are addressed and changes are implemented throughout the program*

Nationwide teleconference meetings provided the ambassadors with a way to collaborate on innovative ideas and to identify ongoing challenges faced by the program on a national level. Increasing attendance at events was one of the major challenges encountered. The LearnENT ambassadors learned that students interested in OHNS as a career choice comprise a small number of medical students. As such, to combat the attendance challenge, advertisement of LAP events was catered towards students interested in a variety of medical specialties including surgery, family practice, pediatrics, and emergency medicine. Ambassadors were also encouraged to host LAP events at times when students are studying OHNS in their curricula such as during their OHNS block in pre-clerkship. These suggestions were made explicit to students through both regular email updates and teleconference meetings.

This article provides valuable insight into the benefits of creating a student ambassador program not only with respect to user engagement, but also in building a strong, collaborative community. In the future, the LAP model may also be implemented and used across multiple specialties for different medical education initiatives. Furthermore, student-led ambassador programs have the potential to increase the awareness and usage of learning resources, particularly in smaller, underrepresented disciplines like OHNS. With medical students involved in these medical education initiatives in different medical schools across Canada, the dissemination of knowledge and national involvement is more likely.
